# COVID-19: an ethical predicament and the dichotomy of society

**DOI:** 10.1136/postgradmedj-2020-139537

**Published:** 2021-01-15

**Authors:** Harkirat Singh Talwar, Tushar Aditya Narain

**Affiliations:** All India Institute of Medical Sciences, Rishikesh, Uttarakhand, India; Department of Urology, University College London Hospitals NHS Foundation Trust, London, UK

Waiting patiently to get myself tested for COVID-19, several thoughts crossed my mind; Did I sign up for this? Do I risk my safety for others? Is this my moral responsibility? And how did I find myself outside the testing booth? The answer to the last question was that I was a primary suspect in contact with the nursing officer in my department who had tested positive for the dreaded COVID a day before. Although my result was negative and I have been put under quarantine, several questions trouble me. And some go as far back as to why did I step foot into a medical school? Is it all worth it?

Not just me, these are some of the questions facing every healthcare professional working as a frontline warrior battling this deadly pandemic that has befallen mankind. Over 9 months and millions infected, the end seems nowhere in sight. On one hand, we have the adversities and the risks involved at workplace in such trying times; on the other, stories of mistreatment of healthcare workers act as a huge deterrent to our morale and resolve to continue this fight which has uncertainty written all over it.

Refusing rented accommodation for healthcare workers or pelting them with stones when all they were doing were fulfilling their responsibility of isolating the contacts are some of the examples which has put a huge dent into the passion and resolution with which we had decided to join this noble profession.^1^ Am I still the young 17 years old pledging the Hippocratic oath at the top of my voice with all passion and hope? I guess not, 11 years on and having seen numerous instances of ill treatment of medics, I have no qualms in saying that this honourable profession does not enjoy the same admiration and reverence it once did.

And talking about the Hippocratic oath,^2^ we have been taught the concept of primum non nocere, which means first do no harm in Latin. But does this apply only to the patients we cater to? Should not this first apply to ourselves? Should not we be not harming ourselves, mentally or physically? Be it the airline safety protocol or the disaster management protocol, the rule is to always equip yourself before you help others. And that in my opinion can be extrapolated to our current scenario. In all the love and respect for the work we do, we as healthcare professionals forget ourselves, forget our families who despite being thousands of miles away do not proceed with their lives before ensuring our safety first. We owe it to them.

Then the question arises do we treat the society just the way it treats us? The answer is no. As there might be a huge chunk of the community who might have lost the respect for the medics for whatever reasons, I would not go on to the extent of generalising the entire society as thankless. There are still people who immensely revere the medical fraternity also known as the white brigade and have pinned all their hopes on us in these difficult times. We need to work for them. We need to fight for them.

Despite the adversities, this virus has sprung on the human race, if there is one solace the same community at large has, the one belief that they have put their heart into, is the trust they have on us, the medics, the first-line defence. We are supposed to be their heroes. When thousands stood in their balconies clapping for us across the world or when there were songs and tributes written as an ode to our fraternity, it highlighted their vulnerability and how they trusted us to overcome this mayhem and get them across the line.

Borrowing a quote by Nick Fury from the Avengers movie ‘There was an idea to bring together a group of remarkable people, to see if we could become something more’,^3^ I would go on to say that probably God intended that group of people to be us, the medics and the paramedics. And we do hold a moral responsibility to help, to serve, to provide and to heal. And this has put a huge responsibility on the shoulders of the medical fraternity; clinicians, researchers and healthcare workers alike. The front liners are working tirelessly to curb and mitigate the effects of the disease while the researchers are brainstorming behind the scene to find a cure, to find a vaccine which can put an end to all this mayhem.

With the social media and news agencies abuzz with rising numbers and the toll the virus has taken worldwide, it is very easy to fall prey to rumours and may lead to an increase in panic, anxiety and apprehension.^4^ This has given rise to an increase in the mental health problems, not just in the general population but the healthcare personnel which can further cloud their resolve to fight.^5^ Also, it is very essential to keep a clear head moving forward which can be achieved by staying connected, fighting as a team and keeping all negative thoughts at bay.

Thus at present, the situation we find ourselves in is akin to those soldiers and military personnel protecting the borders from foreign invasion and despite the bicameral attitude of the society towards its caregivers, we will have to continue marching forward with all precautions ensuring our safety. Coming back to the problem at hand, the COVID-19 pandemic, despite the hardships and risks we face, be it the society we live in or the lack of proper safety equipment at workplace, I hope that we as healthcare providers would not back down from the war we face against the virus and will come out triumphant. And if we are going to win this war, some of us might have to lose a battle or two and in the end it will all be worth it. The noble profession has already started to regain its lost glory and you Mr. SARS CO-V 2 will lose.

We as healthcare professionals often find yourselves in the midst of many ethical dilemmas throughout our career, and the ongoing COVID-19 pandemic is one such situation. We on one hand have our moral and ethical responsibility to help the society in these difficult times and on the other are worried about our own safety and the constant fear of contracting the disease ourselves.^5^ The dichotomous attitude of the society only adds to the predicament. Therefore, we need to downplay the pessimism surrounding us and have to keep marching forward with a clear mind and a positive attitude in our quest to mitigate the effects of the virus.
